# Keratocyte Gene Expression Shaped by ECM Dimensionality: Evidence for Enhanced Quiescence in 3D Culture

**DOI:** 10.3390/bioengineering13080876

**Published:** 2026-07-30

**Authors:** Kara Poole Acevedo, Miguel Miron-Mendoza, Walter Matthew Petroll

**Affiliations:** 1 Department of Ophthalmology, UT Southwestern Medical Center, Dallas, TX 75390, USA; 2 Department of Biomedical Engineering, UT Southwestern Medical Center, Dallas, TX 75390, USA

**Keywords:** cornea, stroma, keratocyte, 3D cell culture, bulk RNA-seq, ECM

## Abstract

In addition to soluble biochemical factors, biophysical cues from the extracellular matrix (ECM) can play an integral role in regulating cell behavior. Previous work has demonstrated that varying ECM dimensionality (3D vs. 2D) and structure (fibrillar vs. non-fibrillar) can produce distinct cell phenotypes; however, the transcriptional changes underlying how cells sense and respond to these different environments are not well understood. Here, we used bulk RNA sequencing to compare the gene expression profiles of primary rabbit corneal keratocytes cultured on collagen-coated substrates and on top of or embedded within fibrillar collagen matrices under serum-free conditions. Differential expression and functional analyses revealed distinct transcriptional profiles across the culture conditions and identified differentially expressed genes and enriched signaling pathways primarily related to the ECM, cell–matrix interactions, cell mechanics, and proliferation. Cells cultured in 3D fibrillar conditions exhibited gene expression patterns that better reflected the normal in vivo keratocyte phenotype, including broad suppression of proliferation and ECM synthesis-related genes. In contrast, cells cultured on 2D substrates showed higher expression of genes associated with an activated phenotype. Overall, these findings demonstrate the strong influence of biophysical cues from the ECM on keratocyte gene expression and highlight the importance of selecting physiologically relevant in vitro models for studies of corneal cell biology, wound healing, and regenerative therapies.

## 1. Introduction

In vitro cell culture is a foundational tool in cell biology, tissue engineering, and therapeutic testing. Traditionally, cells are grown as two-dimensional (2D) monolayers on flat, rigid substrates such as plastic or glass; however, most cells exist in a more complex extracellular environment in vivo. Three-dimensional (3D) culture systems are believed to better approximate an extracellular matrix (ECM) structure that is similar to what cells experience in vivo, by allowing cells to grow within natural (collagen or other ECM analogs) or synthetic matrices or as scaffold-free spheroid systems [1,2,3]. Previous studies have shown that ECM dimensionality can have profound effects on cell–cell and cell–matrix interactions, nutrient and oxygen gradients, mechanical cues, and morphological constraints, which in turn influence cell phenotype and behavior [1,4,5,6,7].

2D culture models remain popular because of their simplicity, reproducibility, and ease of access for microscopy, biochemical assays (e.g., Western Blot and qPCR), and high-throughput techniques (e.g., RNA sequencing). However, 2D culture imposes artificial constraints, as cells are forced to adopt flattened morphologies, and the absence of 3D ECM limits cell–matrix interactions and signaling [8]. 3D culture models are believed to better recapitulate certain in vivo-like conditions because they allow for more physiological cell–cell and cell–matrix interactions and allow for mechanical feedback from the ECM [9].

Numerous studies have demonstrated that cells cultured in 3D show distinct phenotypes relative to their 2D counterparts. For example, cancer cells in 3D exhibit altered morphology, migratory behavior, proliferation kinetics, and drug resistance compared to 2D cultures [8,9,10,11,12,13]. Despite these previous observations of phenotypic and functional differences, few studies have carried out comprehensive gene expression profiling comparing 2D and 3D culture. Comparative studies in cancer and other cell types have used RNA sequencing (RNA-seq) and microarrays to demonstrate that 3D culture, primarily using spheroids or synthetic matrices, can shift transcriptomes toward more in vivo-like gene expression signatures related to ECM, hypoxia, metabolism, and signal transduction [1,8,9,10,14,15,16,17]. However, transcriptional information on cells interacting with native fibrillar collagen matrices is limited. Furthermore, similar transcriptional comparisons for corneal keratocytes in 2D and 3D culture are lacking, as previous studies have focused primarily on phenotypic differences (e.g., morphology, ECM deposition, motility) [18,19,20]. This lack of transcriptional profiling limits our understanding of how biophysical cues from 2D and 3D environments modulate gene regulatory networks and keratocyte fate, and the mechanisms through which keratocytes sense and respond to changes in their extracellular environment.

Corneal stromal cells, or keratocytes, play a critical role in maintaining corneal transparency and mediating the stromal wound healing response following injury or surgery. In their normal non-activated state, keratocytes secrete and organize normal stromal collagens and proteoglycans to maintain the ECM composition and structure required for corneal transparency and mechanical stability [21,22]. After stromal injury or surgery, the release of pro-inflammatory cytokines and growth factors results in the activation of keratocytes towards a migratory fibroblast and/or fibrotic myofibroblast phenotype [23,24]. These activated stromal cells repopulate the wound and deposit new ECM [25,26]. In physiological conditions, this ECM is remodeled to restore corneal transparency. However, in pathological conditions, dysregulated or protracted cellular responses to wound healing cues can lead to corneal fibrosis and scarring, which are leading causes of blindness worldwide [27]. Recently, our lab and others have investigated the transcriptional profiles of cultured corneal keratocytes, fibroblasts, and myofibroblasts; however, these experiments were performed using only 2D substrates [28,29,30]. In the present study, we compare the global gene expression profiles of rabbit corneal keratocytes cultured in 2D and 3D environments under identical biochemical conditions, using both collagen-coated substrates and fibrillar collagen matrices. By applying bulk RNA-seq and downstream bioinformatic analyses, we identify genes and enriched signaling pathways that are differentially regulated in 2D and 3D culture. We relate these transcriptional changes to previously observed differences in keratocyte phenotype, such as ECM deposition, matrix remodeling, and cell mechanics.

## 2. Materials and Methods

### 2.1. Isolation of Primary Rabbit Corneal Keratocytes

We used a well-established serum-free primary culture model that is known to produce a pure population of corneal keratocytes that mimic key aspects of in vivo corneal keratocytes in terms of morphology, cytoskeletal organization, and gene and protein expression [31,32,33,34,35,36,37,38,39]. Normal rabbit keratocytes (NRKs) were isolated from New Zealand white rabbit eyes (Pel-Freez Biologicals, Rogers, AR, USA) and maintained in serum-free (SF) media according to previously described protocols [40]. To summarize, Pel-Freez eyes were placed in RPMI-1640 Medium (Sigma, St. Louis, MO, USA) supplemented with 1% antibiotic antimycotic solution (Sigma) prior to corneal dissection. The epithelium was removed by swabbing with an alcohol pad and scraping with a surgical blade (no. 11; Sigma). Corneal buttons were excised, placed in fresh RPMI solution, and the endothelium was removed by gently scraping with a disposable scalpel (no. 15; Fisher Scientific, Waltham, MA, USA). The corneal buttons were incubated overnight at 37 °C in digestion media consisting of 1× MEM (Gibco, Grand Island, NY, USA) with 2.0 mg/mL collagenase (Gibco), 0.5 mg/mL hyaluronidase (Worthington Biochemical Corporation, Lakewood, NJ, USA), and 2% antibiotic antimycotic solution. Isolated keratocytes were centrifuge-pelleted (4 min at 1500 rpm), resuspended in SF media, and plated in 25 cm^2^ tissue culture flasks. Defined SF media contained Dulbecco’s modified Eagle’s medium (DMEM; Sigma) supplemented with 100 μM non-essential amino acids (Gibco), 100 μg/mL ascorbic acid (Sigma), 1% RPMI vitamin solution (Sigma), and 1% antibiotic antimycotic solution [38]. Cells were maintained in SF media for five days prior to use in experiments.

### 2.2. 2D and 3D Cell Culture

In all experiments, first-passage NRKs were plated with SF media (day 0) and cultured for six days before isolating RNA or fixing samples for fluorescence microscopy. SF media was replenished on days 2 and 5. For 2D culture, NRKs were plated on the surface of collagen-coated substrates or fibrillar collagen matrices, and 3D culture NRKs were embedded within fibrillar collagen matrices. For 2D collagen-coated conditions (2DC), MatTek dishes (MatTek, Ashland, MA, USA) were covered with a neutralized solution of 50 μg/mL type I collagen, incubated for 30 min at 37 °C, then rinsed twice with DMEM before plating NRKs at a density of 50,000 cells/mL (~15,000 cells/cm^2^). For the 2D fibrillar collagen condition (2DF), 300 μL of neutralized collagen solution (1.5 mg/mL) was allowed to polymerize for 90 min at 37 °C before plating NRKs at a density of 50,000 cells/mL (~15,000 cells/cm^2^). For the 3D fibrillar collagen condition, 300 μL of neutralized collagen solution (1.5 mg/mL) containing 100,000 cells/matrix (3DF) was allowed to polymerize for 90 min at 37 °C before adding SF media [32]. To assess the effects of cell density, matrices containing 50,000 cells/matrix (3DF_50) were also plated. For all fibrillar collagen matrices, the 300 μL aliquots of collagen solution were spread over the 20 mm diameter microwells of the Mattek dishes. Neutralized collagen solution was prepared by mixing type I bovine collagen (Advanced BioMatrix, Carlsbad, CA, USA) with 10× MEM, 0.1 N NaOH, and DMEM (with cells for 3D conditions only) to obtain the final concentrations of 50 μg/mL (for 2DC) or 1.5 mg/mL (for 2DF, 3DF, and 3DF_50). A diagram of the 2D and 3D culture conditions demonstrating the position of cells relative to the collagen-coated dish or collagen matrix can be found in Figure S1.

### 2.3. Total RNA Isolation and Sequencing

Total RNA was collected for five different experimental conditions using the Aurum Total RNA Mini Kit (Bio-Rad, Hercules, CA, USA). First, culture dishes were rinsed twice with sterile phosphate-buffered saline (PBS). For all conditions with fibrillar collagen matrices (2DF, 3DF, and 3DF_50), matrices were first lifted from the culture dishes using a small spatula and placed in sterile 2 mL microcentrifuge tubes (3–6 matrices per tube). To isolate the NRKs from the collagen matrix, 1 mL of digestion media was added to each tube and incubated on a rocker for 15 min at 37 °C. The cells were then centrifuge-pelleted (4 min at 1500 rpm), rinsed once with PBS, then resuspended in lysis buffer. For the 2D collagen-coated condition (2DC), culture dishes were incubated with digestion media for 15 min at 37 °C. The dishes were then rinsed with PBS, followed by the addition of lysis buffer. To assess the effects of the digestion media incubation, lysis buffer was added directly to the culture dish (without digestion media) in another group (2DC_L). To collect a sufficient amount of RNA for bulk RNA-seq, 5–6 dishes were pooled for collagen-coated conditions, and a minimum of 11 matrices were pooled for fibrillar collagen conditions. For all conditions, lysates were collected in sterile 1.5 mL microcentrifuge tubes, then mixed with RNase-Free 70% ethanol. RNA isolation steps proceeded according to the manufacturer’s protocol, and total RNA was eluted from the Aurum RNA-Binding Mini Columns using molecular biology-grade water.

RNA concentration and integrity were evaluated using a Thermo Scientific NanoDrop One^C^ (Waltham, MA, USA). For each condition, samples from three separate experiments (biological replicates) collected as detailed above were sent to Novogene Co. (Sacramento, CA, USA) for bulk RNA-seq. Novogene Co. carried out sample quality control (Agilent 5400, Santa Clara, CA, USA) followed by library preparation, quality control, and sequencing using the NovaSeq 6000 platform (Illumina, San Diego, CA, USA). After data quality control, sequencing reads were aligned to the oryCun2 reference genome (HISAT2 version 2.0.5) and gene expression level analysis was conducted (featureCounts version 1.5.0-p3). The original data presented in this study are openly available in NCBI’s Gene Expression Omnibus via the GEO series accession number GSE337743.

### 2.4. Principal Component, Differential Expression, and Enrichment Analyses

Additional bioinformatics analysis was conducted using NovoMagic, a free Novogene platform for data analysis. Principal component analysis (PCA) was performed on the normalized gene expression values (FPKM) of all samples to evaluate differences between the five experimental conditions (2DC, 2DC_L, 2DF, 3DF and 3DF_50). Differential expression analysis was performed for pairwise comparisons of both the three primary conditions (2DC, 2DF, 3DF) and all five conditions (2DC, 2DC_L, 2DF, 3DF, 3DF_50) (DESeq2 version 1.20.0). Parametric analysis of variance (ANOVA) with Benjamini–Hochberg False Discovery Rate correction at *p* = 0.05 was performed on normalized data to identify significant differentially expressed genes (DEGs) with a |log_2_[fold change]| ≥ 1 and an adjusted *p* value ≤ 0.05. KEGG (Kyoto Encyclopedia of Genes and Genomes) enrichment analysis was also carried out for each differential gene set to determine which biological functions and signaling pathways were significantly associated with DEGs (clusterProfiler version 3.8.1). KEGG pathways with an adjusted *p* value < 0.05 were considered significant.

All bar plots, volcano plots, and heatmaps displaying results from differential expression and pathway analyses were created using GraphPad Prism (version 10.5.0). Heatmaps used normalized, log_2_-transformed FPKM values (log_2_[FPKM + 1]) to display relative expression levels across samples (columns) for sets of differentially expressed genes (rows). Hierarchical clustering was carried out on the gene sets so genes and samples with similar expression patterns would be gathered together.

### 2.5. Fluorescence Microscopy

Additional samples from each experimental condition were stained with phalloidin to visualize cell morphology and F-actin organization. Briefly, cells were fixed with 3% paraformaldehyde for 15 min, washed in PBS (3 times, 10 min each), then permeabilized with 0.5% Triton X-100 for 15 min. For F-actin labeling, cells were incubated with Alexa Fluor 633 Phalloidin (Invitrogen, Eugene, OR, USA) for 2 h at 37 °C, then washed in PBS (3 times, 20 min each). To label cell nuclei, cells were incubated with DAPI (Invitrogen) for 20 min at room temperature, then rinsed twice. Fluorescence images were acquired using a laser confocal microscope (Leica SP8, Heidelberg, Germany) and a 25× water immersion objective. A stack of optical sections (z-series) was acquired for each image using a step size of 2 μm. Image processing was carried out using ImageJ (version 1.54). Summed voxel projections were made for each channel, then combined using the merge channels function.

## 3. Results

### 3.1. Keratocyte Morphology and Transcriptional Profiles in 2D and 3D Culture

**Figure 1:**
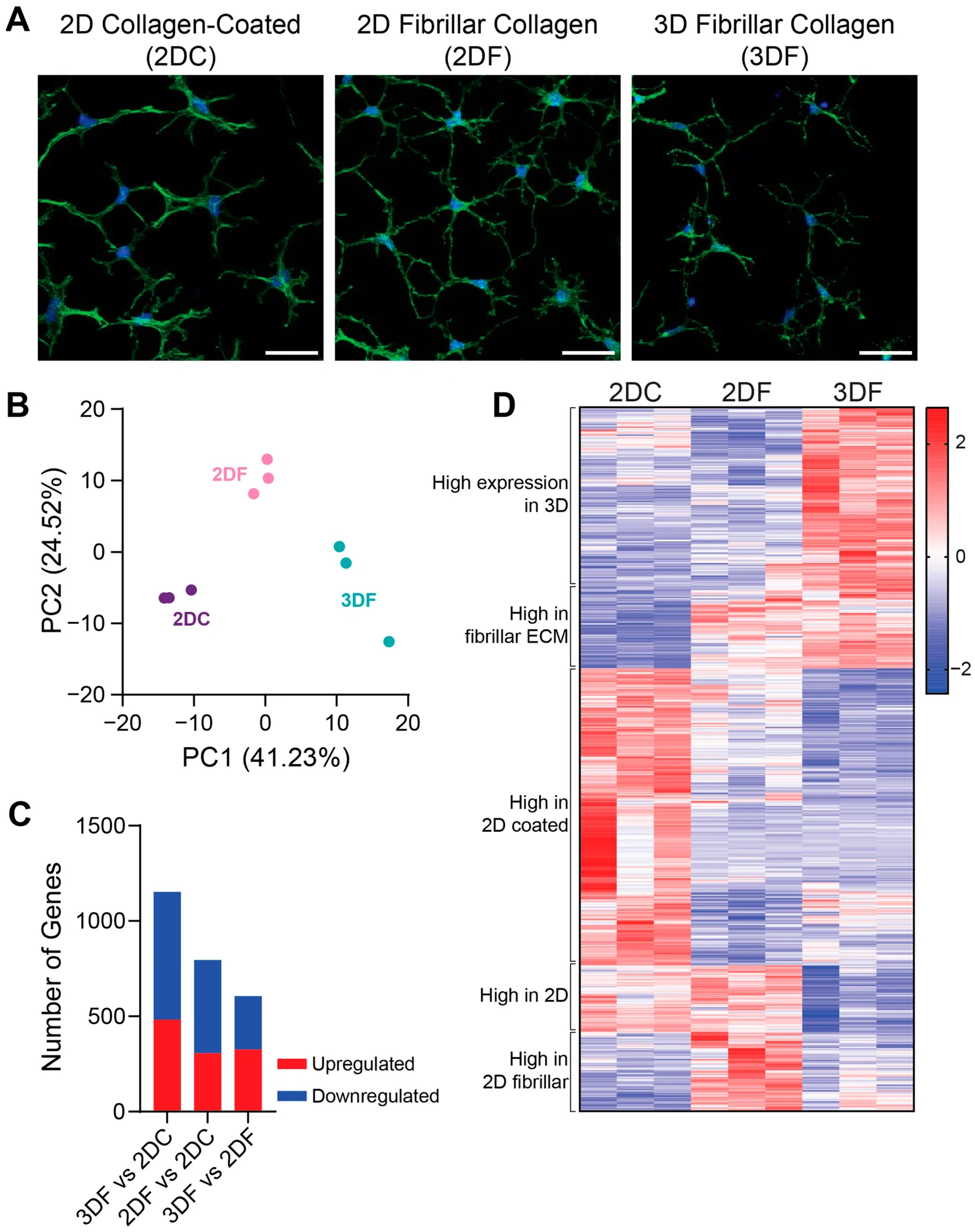
Corneal keratocytes in serum-free culture adopted distinct morphologies and transcriptional profiles in 2D and 3D environments. (**A**) Representative confocal images of keratocytes cultured on top of collagen-coated substrates (2DC), on top of fibrillar collagen matrices (2DF), or embedded within fibrillar collagen matrices (3DF). Samples were fixed and labeled for F-actin (green) and DAPI (blue). Scale bar is 50 μm. (**B**) Principal component analysis (PCA) was performed on all samples (*n* = 3 biological replicates) from the 2DC, 2DF, and 3DF conditions using normalized, log_2_-transformed FPKM values. PC1 and PC2 accounted for 41.23% and 24.52% of the variance, respectively. (**C**) Bar plot displaying the number of significant upregulated (red) and downregulated (blue) differentially expressed genes (DEGs) for pairwise comparisons of the three experimental conditions. DEGs with an adjusted *p* value ≤ 0.05 and |log_2_[fold change]| ≥ 1 were considered significant. (**D**) Heatmap showing the relative expression of each significant DEG (rows) across the three conditions (columns). Relative expression is based on normalized, log_2_-transformed FPKM values (log_2_[FPKM + 1]). Red color indicates high relative expression and blue color indicates low. The differential gene set includes all DEGs from the pairwise comparisons in (**C**).

Keratocytes cultured in 2D and 3D environments developed different morphologies while being maintained in serum-free media (Figure 1A). While cells in all ECM conditions developed a similar stellate morphology that is characteristic of corneal keratocytes cultured in serum-free media [41], cells on top of (2DF) or embedded within (3DF) fibrillar collagen matrices had somewhat thinner cell extensions with more branching and punctate F-actin labeling than cells on 2D collagen-coated substrates (2DC). Due to the lower effective cell density in the 3D conditions, cells in 2D exhibited greater numbers of cell–cell contacts.

To investigate the impact of different ECM environments at the transcriptional level, three samples of total RNA were collected for each experimental condition and submitted for bulk RNA-seq. The RNA isolation method optimized for this study yielded a suitable quantity (>200 ng) and quality (RIN > 9.0) of RNA from cells cultured in 2D and 3D environments. Sequencing quality and read mapping results were also evaluated, and all downstream analyses were performed based on the clean, high-quality data (Tables S1 and S2). Principal component analysis (PCA) was performed to evaluate differences in the overall gene expression profiles of each of the samples (Figure 1B). The PCA plot showed clear separation between the 2D collagen-coated (2DC), 2D fibrillar collagen (2DF), and 3D fibrillar collagen (3DF) conditions, while samples within each condition were clustered together.

Differential expression analysis revealed that comparing cells in 3D fibrillar collagen to those on 2D collagen-coated substrates resulted in the highest number of significant DEGs (1153; Figure 1C). This was followed by the comparisons of 2D fibrillar collagen vs. 2D collagen-coated (795 DEGs), then 3D vs. 2D fibrillar collagen (605 DEGs). The combined differential gene set from these three comparisons contained 1655 DEGs. The relative expression heatmap for this differential gene set showed several groups of genes with distinct patterns of expression across the three experimental conditions (Figure 1D). A large set of genes had high expression in the 2D collagen-coated condition (2DC) relative to both fibrillar collagen conditions (2DF, 3DF), while another set showed high expression in fibrillar collagen relative to collagen-coated substrates. Gene sets with high relative expression in 2D (2DC, 2DF) compared to 3D, or with high expression in 3D relative to 2D, were also identified in this heatmap. A gene set with high relative expression in 2DF further distinguished this condition from the others. Thus, both ECM dimensionality (3D vs. 2D) and structure (fibrillar vs. non-fibrillar) impacted the pattern of keratocyte gene expression.

**Figure 2:**
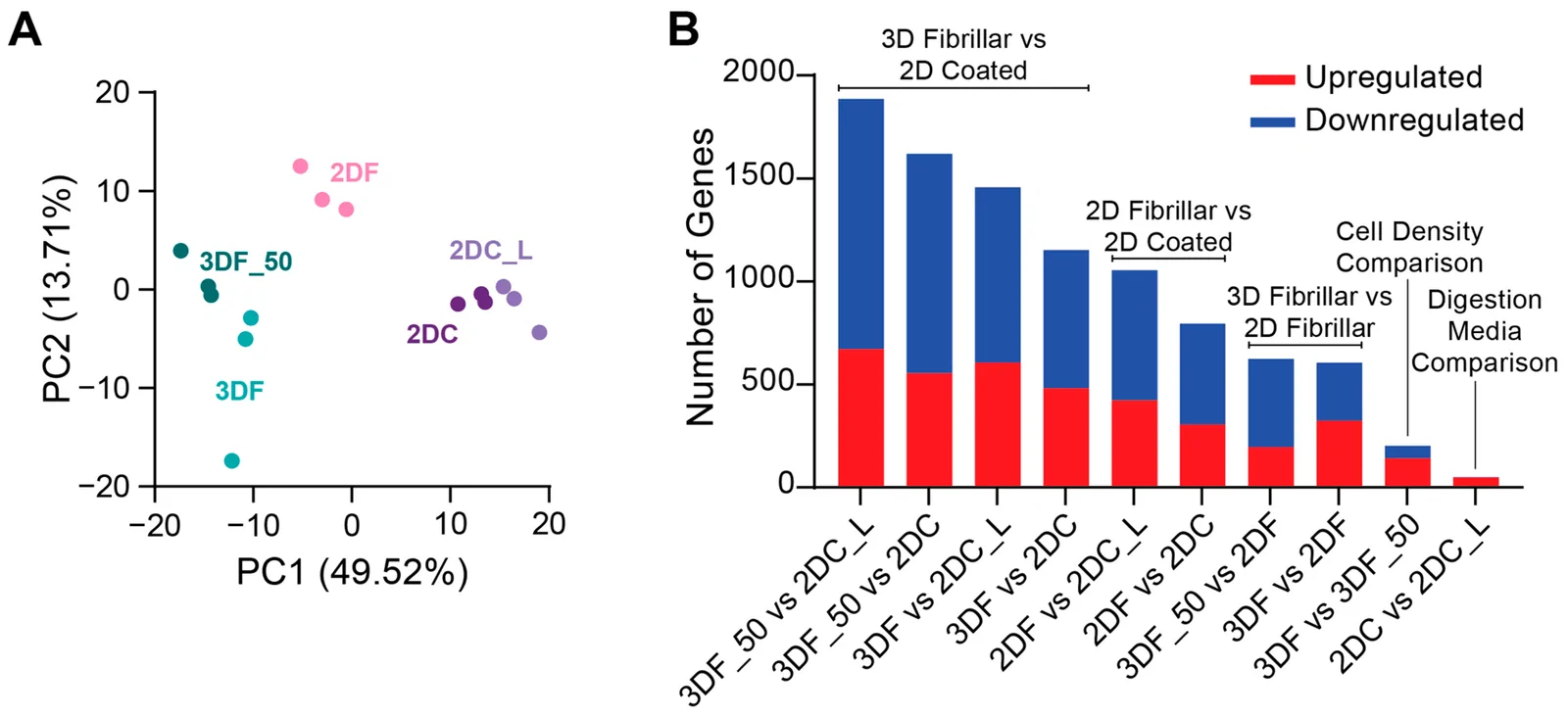
(**A**) Principal component analysis (PCA) was performed on all samples (*n* = 3 biological replicates) from all five experimental conditions using normalized, log_2_-transformed FPKM values. PC1 and PC2 accounted for 49.52% and 13.71% of the variance, respectively. (**B**) Bar plot displaying the number of significant upregulated (red) and downregulated (blue) differentially expressed genes (DEGs) for each pairwise comparison. DEGs with an adjusted *p* value ≤ 0.05 and |log_2_[fold change]| ≥ 1 were considered significant.

Similar observations were made regarding the principal component and differential expression analyses when performed for all five conditions (Figure 2). Changing the cell density or omitting the digestion media incubation had the smallest effects on differential gene expression. In the PCA plot, the two 3D conditions clustered together (3DF, 3DF_50) and the two collagen-coated conditions clustered together (2DC, 2DC_L) indicating similar gene expression profiles (Figure 2A). From the differential expression analysis, 3DF vs. 3DF_50 had only 203 DEGs, many of which were related to cell–ECM interactions, and 2DC vs. 2DC_L had only 50 DEGs, which were mainly associated with the early cellular response to external stimuli and the regulation of cell stress response (Figure 2B and Figure S2). The differential gene set generated from all ten pairwise comparisons consisted of 2876 genes. The corresponding relative expression heatmap showed several sets of genes with distinct patterns of expression across the five conditions, once again distinguishing 3D conditions from 2D, and fibrillar from non-fibrillar (Figure S3). Given the small difference in gene expression between 2DC vs. 2DC_L, and 3DF vs. 3DF_50, we continued to focus our analysis on three of the five experimental conditions—2DC, 2DF, and 3DF—so that we could more directly compare the effects of ECM structure and dimensionality.

### 3.2. Expression of Common Markers for Quiescent and Activated Cells

**Figure 3:**
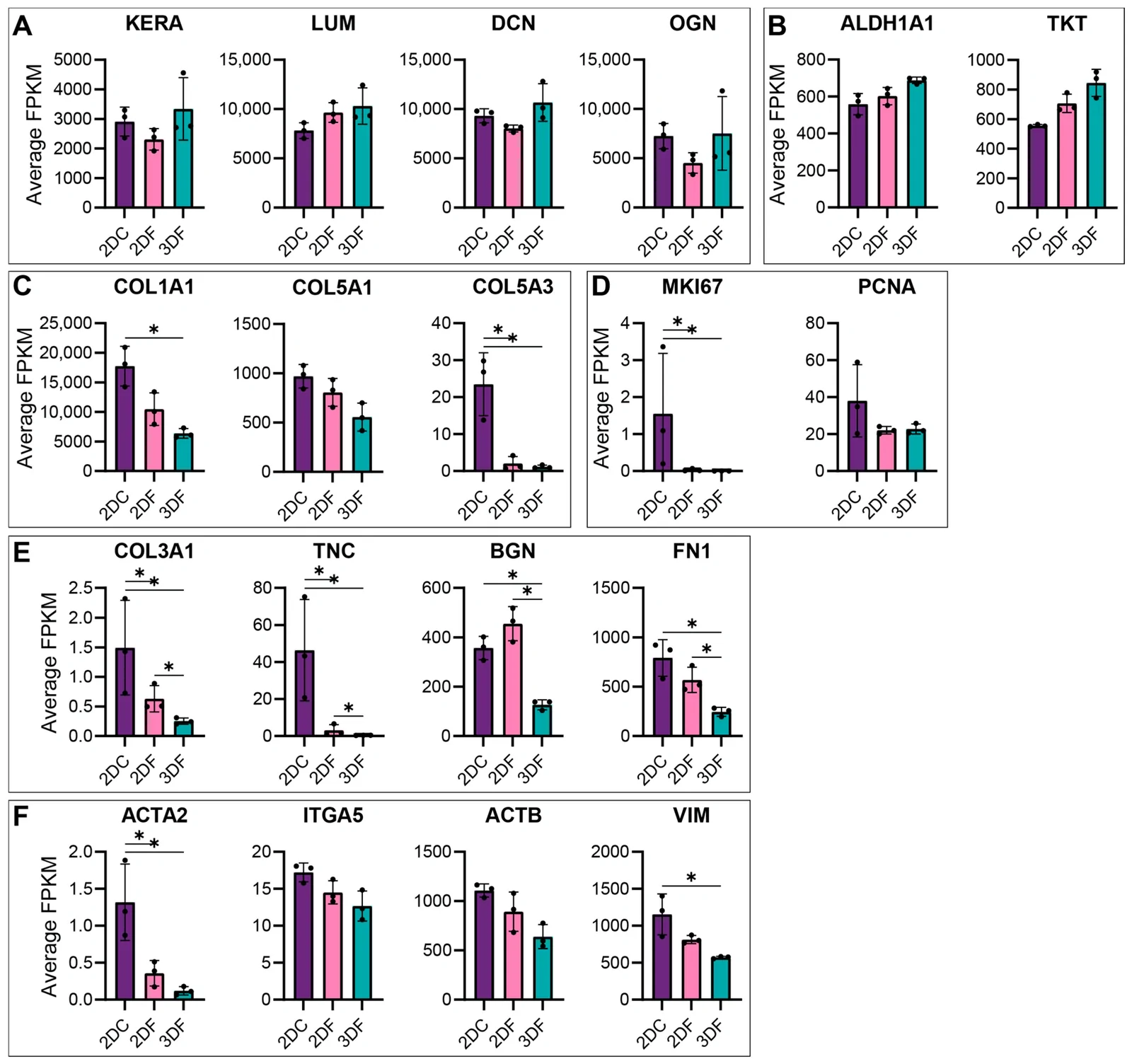
Gene expression levels in 2DC, 2DF, and 3DF conditions for common markers of normal keratocytes (**A**–**C**) and activated stromal cells (**D**–**F**). Bar plots of average FPKM values (*n* = 3 biological replicates) for genes encoding (**A**) proteoglycans, (**B**) crystallins, and (**C**) collagens present in the native corneal stroma. (**D**) Genes associated with cell proliferation. (**E**) ECM- and (**F**) cytoskeleton-related genes that are commonly associated with activated cells and/or fibrosis. Error bars represent standard deviation and asterisks indicate if a gene was found to be significantly differentially expressed between the two indicated conditions based on differential expression analysis (adjusted *p* value ≤ 0.05 and |log_2_[fold change]| ≥ 1).

NRKs cultured in 2DC, 2DF, and 3DF conditions showed similarly high levels of expression for genes that are commonly associated with normal keratocytes. Genes encoding native stromal proteoglycans such as keratocan (KERA), lumican (LUM), decorin (DCN), and mimecan (OGN), as well as the corneal crystallins aldehyde dehydrogenase 1 family member A1 (ALDH1A1) and transketolase (TKT), did not show significant differential expression in the comparisons of these three conditions (Figure 3A,B) [22,33,37,42]. However, these genes tended to have the highest expression levels for cells in 3D relative to the 2D conditions. In contrast, genes encoding type I and type V collagens, the primary fibrillar collagens that make up the corneal stroma [22,43], had the lowest expression levels in the 3D condition (Figure 3C). Gene expression for collagen type I alpha 1 chain (COL1A1) was significantly downregulated in 3DF as compared to 2DC, and collagen type V alpha 3 chain (COL5A3) was significantly downregulated in both 2DF and 3DF compared to 2DC. Collagen type V alpha 1 chain (COL5A1) was not significantly differentially expressed; however, it followed a similar pattern with 2DC having the highest expression levels, followed by 2DF, then 3DF.

Several genes that are commonly associated with activated stromal cells or fibrosis were differentially expressed when comparing NRKs cultured in 2D and 3D environments. Marker of proliferation Ki-67 (MKI67) and proliferating cell nuclear antigen (PCNA), two genes involved in cell proliferation [39,44,45,46], both showed lower expression in 2DF and 3DF relative to 2DC (Figure 3D). Genes encoding ECM components associated with fibrosis and wound healing, such as type III collagen (COL3A1) [47,48], tenascin C (TNC) [49,50,51], biglycan (BGN) [47,52], and fibronectin (FN1) [48,53], were significantly downregulated in 3D as compared to both 2D conditions (Figure 3E). COL3A1 and TNC also showed significant differential expression when comparing the two 2D conditions, with expression levels being downregulated in 2DF relative to 2DC. Genes encoding cytoskeletal components that typically show increased expression in activated stromal cells, including alpha-smooth muscle actin (α-SMA; ACTA2) [38,54], integrin subunit alpha 5 (ITGA5) [36,47,55], beta actin (ACTB) [36,56], and vimentin (VIM) [57], showed a similar trend where 2DC had the highest expression and 3DF had the lowest (Figure 3F). As expected for serum-free culture, ACTA2 expression levels were very low for all three conditions; however, its expression was significantly downregulated in both 2DF and 3DF as compared to 2DC. Overall, the gene expression levels for these common markers of normal keratocytes and activated stromal cells appear to point toward a more quiescent transcriptional profile for cells cultured in 3D fibrillar collagen.

### 3.3. Pathway Enrichment Analysis

**Figure 4:**
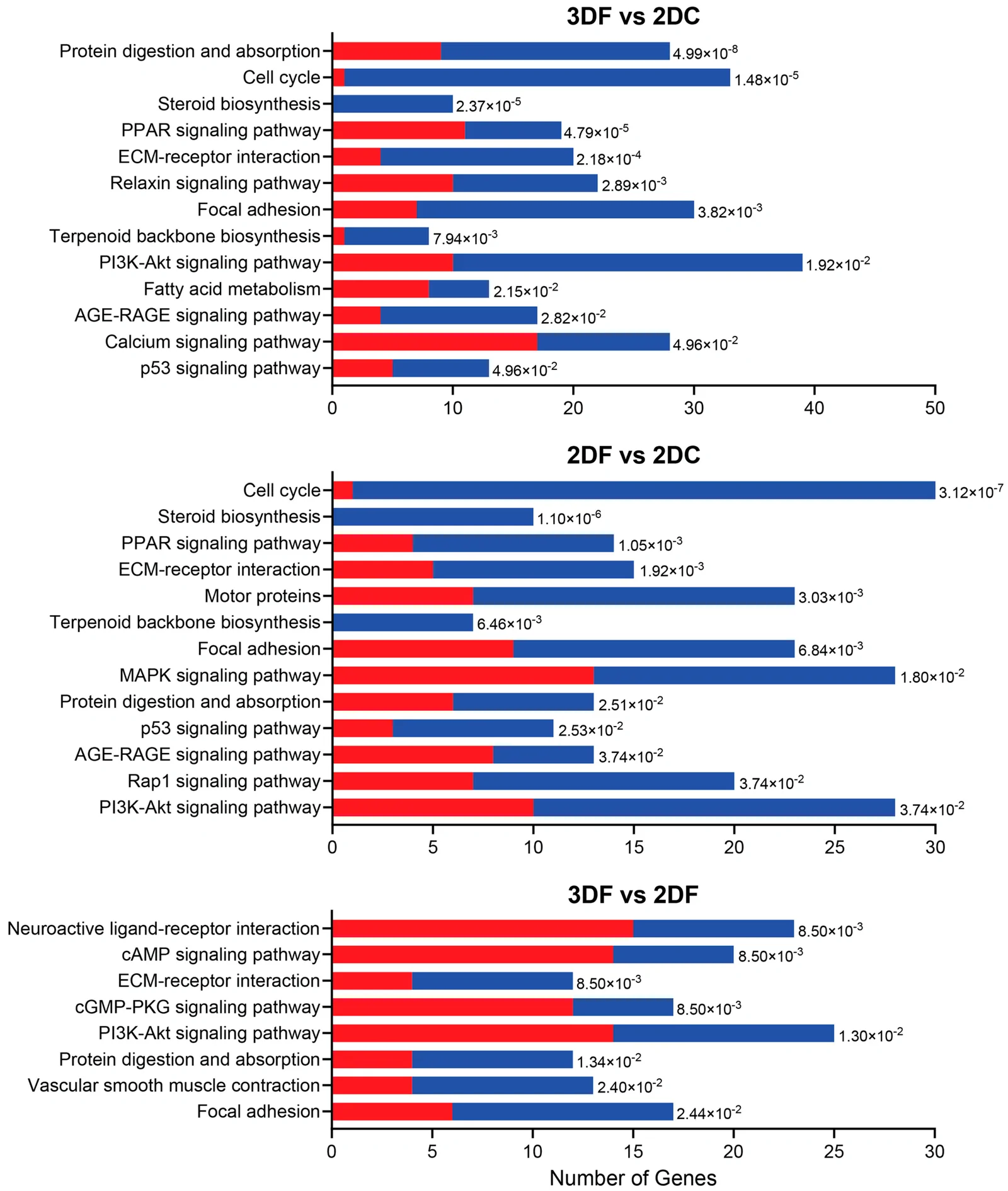
KEGG enrichment analysis for 3DF vs. 2DC, 2DF vs. 2DC, and 3DF vs. 2DF. Bars represent the number of upregulated (red) or downregulated (blue) genes in each significant pathway shown (adjusted *p* value < 0.05). Adjusted *p* values for each pathway are indicated to the right of the bar.

KEGG enrichment analysis was performed for the comparisons of 3DF vs. 2DC, 2DF vs. 2DC, and 3DF vs. 2DF to identify significantly enriched biological functions and signal transduction pathways associated with DEGs (Figure 4). Overall, pathways related to cell–ECM interactions, ECM maintenance, cell adhesion, motility, proliferation, and metabolism were significantly enriched for these comparisons. For example, the ECM–receptor interaction, focal adhesion, and protein digestion and absorption pathways were significantly enriched in all three comparisons. This suggests that shifting from a 3D to a 2D culture system, or from a fibrillar to non-fibrillar ECM, significantly altered the expression of genes involved in cell adhesion, ECM synthesis, and remodeling. The cell cycle and PI3K-Akt signaling pathways were significantly enriched for the comparisons of 3DF vs. 2DC and 2DF vs. 2DC. Genes in these pathways were primarily downregulated in these comparisons, indicating that cells in fibrillar collagen conditions may be less proliferative compared to those on collagen-coated substrates.

### 3.4. Differentially Expressed Genes of Interest

**Figure 5:**
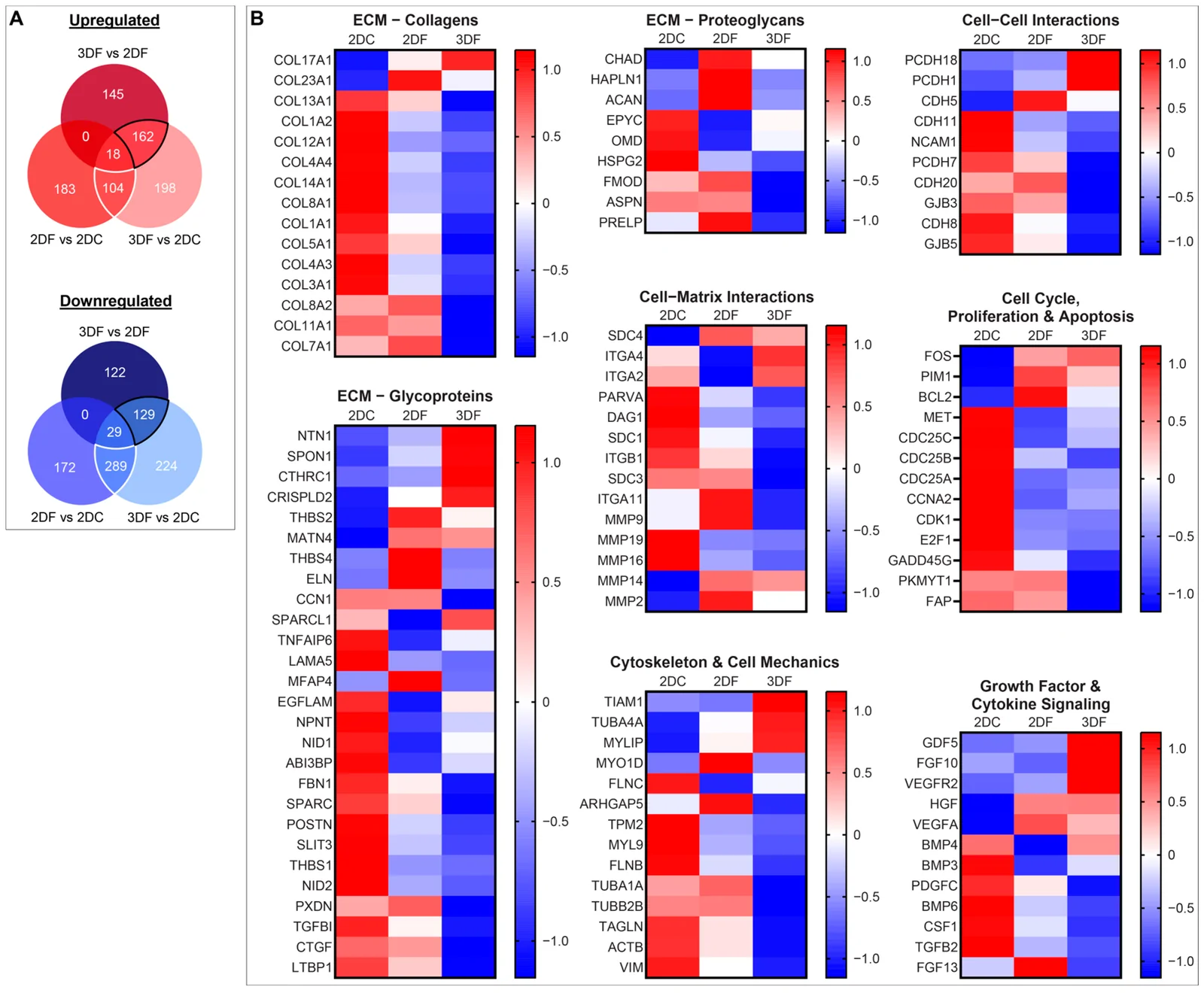
(**A**) Venn diagrams showing the number of significant DEGs that were upregulated or downregulated in one or more comparison. Overlapping areas outlined in black represent the sets of genes that were differentially expressed in both 3D vs. 2D comparisons (3DF vs. 2DC and 3DF vs. 2DF). Overlapping areas outlined in white represent the sets of genes that were differentially expressed in both fibrillar vs. non-fibrillar comparisons (2DF vs. 2DC and 3DF vs. 2DC). (**B**) Heatmaps of normalized, log_2_-transformed FPKM values for a subset of significant DEGs selected from differential expression and KEGG analyses.

Differential expression analysis for 3DF vs. 2DC, 2DF vs. 2DC, and 3DF vs. 2DF identified many genes that were significantly differentially expressed in more than one comparison. Venn diagrams were created to visualize overlaps in the differential gene sets for these three comparisons, as well as to identify DEGs that were unique to each comparison (Figure 5A). For example, 3DF vs. 2DF had 145 upregulated and 122 downregulated genes that were only differentially expressed in this comparison. The overlapping areas for 3DF vs. 2DF and 3DF vs. 2DC (black outlines in Figure 5A) highlight sets of upregulated (162) and downregulated (129) genes that were likely differentially expressed due to the difference in ECM dimensionality. Sets of genes that were likely differentially expressed due to the presence or absence of fibrillar collagen are highlighted by the overlapping areas for 2DF vs. 2DC and 3DF vs. 2DC (104 upregulated, 289 downregulated, white outlines in Figure 5A).

A subset of significant DEGs of interest were selected from the differential expression and KEGG pathway analyses and were organized into several categories based on their primary roles/functions and the types of proteins they encode. The most prominent categories were ECM, cell–matrix interactions, cell–cell interactions, cytoskeleton and cell mechanics, cell cycle, proliferation and apoptosis, and growth factor and cytokine signaling (Figure 5B). The relative expression heatmaps for these gene sets help to further highlight the overall trends in differential expression and to identify DEGs that distinguish cells in 3D culture from those in 2D, as well as fibrillar from non-fibrillar.

In general, cells in the 2DC condition exhibited the highest levels of expression for ECM-related genes, including an abundance of collagens, glycoproteins, and proteoglycans. Interestingly, genes encoding basement membrane collagens (COL4A1, COL4A3, COL4A4, COL7A1, COL8A1, COL8A2) [58] showed higher expression in 2D compared to 3D. Perlecan (HSPG2), a proteoglycan that is another major ECM component of basement membranes [58,59], was also highly expressed in 2DC compared to both 2DF and 3DF. Several glycoproteins and proteoglycans associated with the regulation of TGFβ activity also had higher expression in 2D conditions, including asporin (ASPN), fibrillin 1 (FBN1), and fibromodulin (FMOD) [60]. Genes that showed higher expression in 3D included two transmembrane collagens (COL17A1, COL23A1) and several glycoproteins (NTN1, SPON1, THBS2) that play a role in cell adhesion [61]. Additional genes encoding glycoproteins involved in tissue development and remodeling that were highly expressed in 3D included SPON1, CTHRC1, CRISPLD2, and MATN4.

Two notable genes involved in cell–matrix interactions that were upregulated in fibrillar collagen conditions were the matrix metalloproteinases MMP2 and MMP14. These MMPs are involved in the breakdown of ECM in normal physiological processes such as development and tissue remodeling, and MMP14 has been shown to be especially important for cell migration and invasion in 3D matrices [62]. In contrast, MMPs that have been linked to wound healing and fibrosis (MMP9, 19, and 16) were more highly expressed in 2D. Genes involved in cell–cell interactions were primarily downregulated in 3D compared to 2D, indicating that there may be fewer cell–cell contacts. This included several genes encoding cadherins (CDH11, CDH20, CDH8) and gap junctions (GJB3, GJB5). Genes involved in cell–cell interactions that were more highly expressed in 3D included protocadherins 1 and 18 (PCDH1, PCDH18), as well as cadherin 5 (CDH5), which was highly expressed in both fibrillar collagen conditions.

Many of the genes related to the cytoskeleton and cell mechanics also tended to be more highly expressed in the 2DC condition. This included several genes involved in the regulation of the actin cytoskeleton (FLNC, TPM2, FLNB, TAGLN), which help to support cell shape, contractility, and migration [63]. Genes that showed higher expression in 3D and may contribute to the regulation of cell shape and motility in this condition included TIAM1, TUBA4A, and MYLIP.

In the cell cycle, proliferation and apoptosis category, genes that were more highly expressed in fibrillar conditions were primarily related to the promotion of cell survival (FOS, PIM1, and BCL2) [64,65]. Key cell cycle regulators, such as cyclins, CDKs, checkpoints and phosphatases, were more highly expressed in the 2DC condition. Many genes related to growth factor and cytokine signaling were also differentially expressed between 2D and 3D conditions. Genes involved in regenerative and developmental signaling were found to be more highly expressed in 3D, while genes with roles in fibrotic and inflammatory signaling were more highly expressed in 2D.

## 4. Discussion

The phenotypic responses of corneal keratocytes to their extracellular environment have been extensively studied in vitro. These previous studies primarily used 2D culture systems, despite keratocytes residing in a complex 3D fibrillar matrix in vivo. ECM dimensionality (3D vs. 2D) and structure (fibrillar vs. non-fibrillar) have been shown to significantly impact cellular morphology, cytoskeletal organization, and cell mechanics; however, the underlying transcriptional changes are not well understood [31,66,67]. In this study, we compared bulk RNA-seq profiles of keratocytes cultured under three conditions: 2D collagen-coated substrates (2DC), 2D fibrillar collagen (2DF), and 3D fibrillar collagen (3DF) to identify transcriptional changes driven by ECM dimensionality and structure.

In serum-free culture, normal rabbit keratocytes (NRKs) are known to express common keratocyte markers and are typically characterized as being mechanically quiescent. In this study, we were able to capture significant differences in gene expression between NRKs cultured in SF media on rigid 2D collagen-coated substrates, on 2D fibrillar collagen matrices, and within 3D fibrillar collagen matrices. These results suggest that even in the “non-activated” state that is characteristic of serum-free culture and normal in vivo conditions, these cells are still sensing and responding to biophysical cues from the extracellular environment on a transcriptional level.

By comparing cells in a 3D fibrillar collagen environment to those on a rigid collagen-coated surface (3DF vs. 2DC), we were able to capture the mixed effects of ECM dimensionality, structure, and stiffness on keratocyte gene expression. Comparing cells in 3D to those on top of fibrillar collagen matrices (3DF vs. 2DF) helped to further isolate dimensionality-driven transcriptional changes because the cells were exposed to the same fibrillar collagen cues (i.e., matrix structure and stiffness is matched), leaving dimensionality as the main variable. Lastly, the comparison of cells cultured on top of fibrillar collagen matrices to those on collagen-coated surfaces (2DF vs. 2DC) helped isolate the effects of matrix structure by comparing a 2D fibrillar collagen environment to a non-fibrillar collagen coating.

From these comparisons, two principal gene expression signatures emerged: dimensionality-dependent (3D vs. 2D) and structure-dependent (fibrillar vs. non-fibrillar). Dimensionality-dependent transcriptional regulation was defined by genes consistently differentially expressed in both 3DF vs. 2DC and 3DF vs. 2DF. Upregulated genes in these comparisons were primarily involved in ECM remodeling, metabolic adaptation, and stress/survival signaling. In contrast, downregulated genes encompassed many core cell cycle regulators (cyclins, CDKs, MCMs, checkpoint genes), and genes related to the cytoskeleton, cell contractility, cell–cell interactions, and ECM synthesis. Together with their related signaling pathways, these DEGs indicate that embedding keratocytes in 3D broadly reinforces a more quiescent, non-proliferative transcriptional state. Dimensionality-driven changes in cell phenotype have previously been demonstrated in studies of corneal keratocytes [32]. Previous work has shown that keratocytes maintain a more native morphology and pattern of ECM deposition when cultured in 3D matrices. For example, Thompson et al. observed that rabbit corneal keratocytes in 3D collagen matrices downregulate wound-healing-related responses compared to cells on 2D surfaces [20]. In addition, human corneal stromal stem cells grown in 3D scaffolds showed upregulation of keratocyte marker genes (KERA, LUM, ALDH3A1, etc.) relative to 2D conditions [68].

Structure-dependent regulation was defined by genes consistently differentially expressed in both 3DF vs. 2DC and 2DF vs. 2DC, highlighting transcriptional responses to fibrillar ECM regardless of if the cells were in a 2D or 3D environment. Upregulated genes pointed to altered cell–ECM interactions (integrin subunits and syndecans) and survival signaling (HGF, VEGFA, BCL2). Genes that appeared to be downregulated in response to fibrillar ECM included basement membrane collagens (COL4A1-5), laminins, fibronectin (FN1), tenascin-C (TNC), and proliferation-associated growth factors (FGF1, PDGFB, IGF1). Across both 2DF and 3DF conditions, keratocytes also consistently downregulated nearly all core cell cycle drivers, including cyclins (CCNA2, CCNB1/2, CCNE2), CDK1, replication factors (MCMs, CDC6, CDC45), and spindle checkpoint regulators (BUB1, BUB1B, CDC20, ESPL1) as compared to the 2DC condition. This pattern suggests that fibrillar ECM is the primary signal involved in the suppression of cell cycle gene expression, while 3D culture further reinforces this transcriptional profile. This finding aligns with the physiological behavior of corneal keratocytes, which are non-proliferative in the uninjured cornea [39]. Overall, these observed trends in differential gene expression suggest that cells exposed to fibrillar ECM suppress proliferation and ECM synthesis pathways while reinforcing cell adhesion and survival signaling.

In general, the transcriptional changes in 3D and fibrillar contexts compared to rigid 2D culture appear to align well with the state of stromal keratocytes observed in vivo. Across all conditions, classical markers of normal keratocytes (KERA, LUM, ALDH1A1) remained robustly expressed, with the highest relative expression levels often seen in the 3DF condition. In contrast, markers of stromal cell activation (ACTA2, POSTN, TAGLN, FN1) exhibited the lowest levels in 3DF, reinforcing that embedding keratocytes in 3D fibrillar ECM produces a transcriptional profile that reflects a more quiescent phenotype. These observations are consistent with prior studies of corneal keratocyte behavior that showed reduced activation when cells were cultured in more biomimetic matrices [69]. These patterns of gene expression also align well with recent single-cell RNA-seq (scRNA-seq) findings from our lab [70]. Many of the genes that were upregulated in 3D culture appear to coincide with genes that were highly expressed in scRNA-seq clusters of keratocytes from normal rabbit corneas (e.g., KERA, DCN, ALDH1A1, TKT), whereas genes that were downregulated in 3D corresponded to those that had higher expression in scRNA-seq clusters of migratory and proliferative fibroblasts from corneas undergoing wound repair (e.g., TNC, BGN, ASPN, CDH11, VIM, MKI67, CDK1, CCNA2).

Although several markers of stromal cell activation and fibrosis were observed to be differentially expressed across our ECM conditions, these genes showed low levels of expression overall due to this study being limited to serum-free culture. Future experiments incorporating growth factors present during wound healing, such as PDGF and TGFβ, could be used to determine the extent to which 3D and fibrillar ECM is able to suppress the expression of these markers. Exploring transcriptional differences due to fibrillar ECM using the 2DF and 2DC conditions also presents a potential limitation of the current study, as these results may be confounded by the difference in stiffness between these two conditions. However, previous studies have demonstrated that NRKs in SF culture do not show significant phenotypic differences when cultured on 2D substrates of different stiffnesses [71]. There is also a lower effective cell density in the 3D condition as compared to the 2D conditions. However, changing the cell density in 3D culture had minimal effects on their transcriptional profile.

## 5. Conclusions

This research provides new insights into the transcriptional differences underlying changes in phenotype observed between keratocytes in 2D and 3D culture. In general, 3D and fibrillar ECM environments appear to impose transcriptional changes that lead to widespread suppression of proliferation and ECM synthesis, while enhancing adhesion, mechanosensory, and survival signaling. The results of this study may help inform in vitro model selection when studying cell response to additional biochemical and biophysical cues, or when testing therapeutics, as cell response is likely to differ in 2D and 3D culture.

## Data Availability

The original data presented in this study are openly available in NCBI’s Gene Expression Omnibus via the GEO series accession number GSE337743.

## Supplementary Materials

The following supporting information can be downloaded at https://www.mdpi.com/article/10.3390/bioengineering13080876/s1. Figure S1. Diagram of the 2D and 3D culture conditions demonstrating the position of cells relative to the collagen-coated dish (2DC and 2DC_L) or collagen matrix (2DF, 3DF, and 3DF_50). Figure S2. Volcano plots represent genes that were significantly up- or downregulated within the comparisons of (A) 3D fibrillar collagen with 100,000 cells/matrix (3DF) vs. 3D fibrillar collagen with 50,000 cells/matrix (3DF_50) and (B) 2D collagen-coated (2DC) vs. 2D collagen-coated lysis buffer control (2DC_L). DEGs with an adjusted P value ≤ 0.05 (horizontal dashed line) and |log_2_[fold change]| ≥ 1 (vertical dashed lines) were considered significant. Figure S3. Heatmap showing the relative expression of each significant DEG (rows) across all five conditions (columns). Relative expression is based on normalized, log_2_-transformed FPKM values (log_2_[FPKM + 1]). Red color indicates high relative expression and blue color indicates low. The differential gene set includes all DEGs from the pairwise comparisons in Figure 2B. Table S1. Summary of sequencing data quality for all samples. Table S2. Summary of read mapping results for all samples.

## Abbreviations

The following abbreviations are used in this manuscript:

2D	Two-dimensional
3D	Three-dimensional
ECM	Extracellular matrix
RNA-seq	RNA sequencing
NRK	Normal rabbit keratocyte
SF	Serum-free
DMEM	Dulbecco’s modified eagle’s medium
2DC	2D collagen-coated
2DF	2D fibrillar collagen
3DF	3D fibrillar collagen
PBS	Phosphate-buffered saline
PCA	Principal component analysis
FPKM	Fragments per kilobase of transcript per million fragments mapped
DEG	Differentially expressed gene
KEGG	Kyoto Encyclopedia of Genes and Genomes
