# Supplementary material for: Keratocyte Gene Expression Shaped by ECM Dimensionality: Evidence for Enhanced Quiescence in 3D Culture
Source: Bioengineering (Basel). 2026 Jul 30;13(8):876. doi: 10.3390/bioengineering13080876 (PMC13509429; doi:10.3390/bioengineering13080876)
Supplement: Supplementary file 1 [file bioengineering-13-00876-s001.zip › bioengineering-4439678-supplementary.pdf]

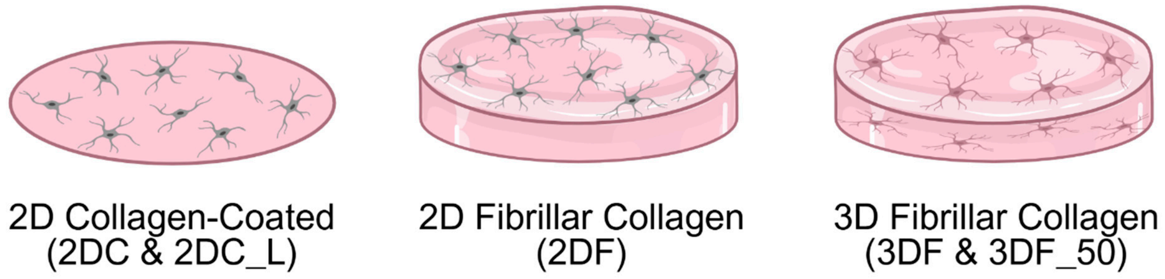

Figure S1. Diagram of the 2D and 3D culture conditions demonstrating the position of cells relative to the collagen-coated dish (2DC and 2DC_L) or collagen matrix (2DF, 3DF, and 3DF_50).

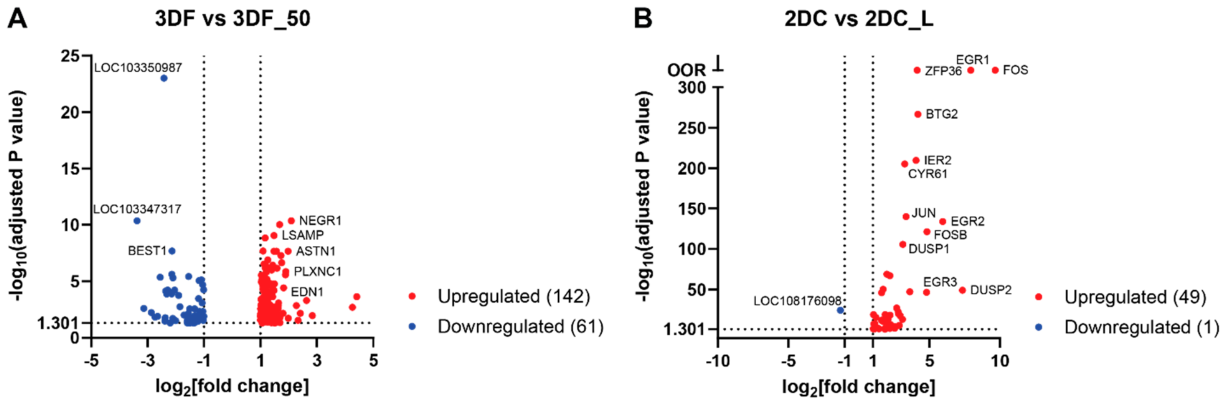

Figure S2. Volcano plots represent genes that were significantly up- or downregulated within the comparisons of **(A)** 3D fibrillar collagen with 100,000 cells/matrix (3DF) vs. 3D fibrillar collagen with 50,000 cells/matrix (3DF_50) and **(B)** 2D collagen-coated (2DC) vs. 2D collagen-coated lysis buffer control (2DC_L). DEGs with an adjusted P value ≤ 0.05 (horizontal dashed line) and $|\log_2[\text{fold change}]| \geq 1$ (vertical dashed lines) were considered significant.

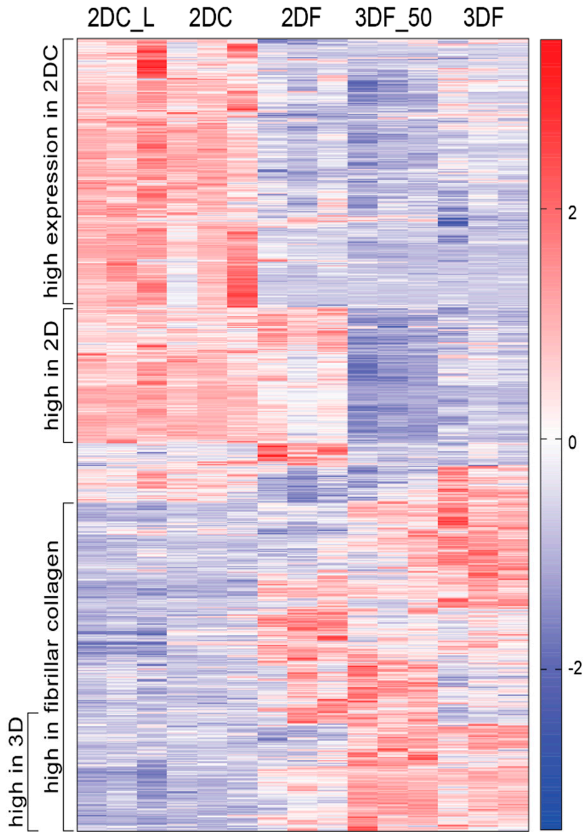

Figure S3. Heatmap showing the relative expression of each significant DEG (rows) across all five conditions (columns). Relative expression is based on normalized, log_2-transformed FPKM values (log_2[FPKM+1]). Red color indicates high relative expression and blue color indicates low. The differential gene set includes all DEGs from the pairwise comparisons in Fig. 2B.

Table S1. Summary of sequencing data quality for all samples.

Culture Condition	Sample Name	Raw Reads	Raw Bases	Clean Reads	Clean Bases	Q20 (%)	Q30 (%)
2DC	E2SF_CMC	98154064	14.72G	96047296	14.41G	97.72	94.12
	E3SF_CMC	73677568	11.05G	71920586	10.79G	97.69	94.06
	E6SF_CMC	69148674	10.37G	67876702	10.18G	97.64	93.86
2DC_L	E2SF_CML	95541554	14.33G	93230116	13.98G	97.75	94.16
	E3SF_CML	92138692	13.82G	90162066	13.52G	97.69	94.04
	E7SF_CML	56103454	8.42G	54804260	8.22G	98.21	95.22
2DF	E10SF_2DF	54912062	8.24G	53588052	8.04G	98.08	94.92
	E5SF_2DF	58046946	8.71G	56203970	8.43G	97.57	93.56
	E9SF_2DF	62538630	9.38G	58993438	8.85G	97.96	94.68
3DF	E6SF_3DF_100	73675318	11.05G	71087308	10.66G	98.06	94.57
	E7SF_3DF_100	65700012	9.86G	63970868	9.6G	98.08	94.9
	E8SF_3DF_100	58310636	8.75G	57208682	8.58G	97.98	94.64
3DF_50	E3SF_3DF_50	46271048	6.94G	44550608	6.68G	98.04	94.85
	E5SF_3DF_50	53506286	8.03G	51239680	7.69G	97.75	94.02
	E7SF_3DF_50	60041926	9.01G	56834002	8.53G	97.96	94.57

Table S2. Summary of read mapping results for all samples.

Culture Condition	Sample Name	Total Reads	Total Mapped	Total Mapped (%)
2DC	E2SF_CMC	96047296	82882876	86.29%
	E3SF_CMC	71920586	62141135	86.40%
	E6SF_CMC	67876702	58944644	86.84%
2DC_L	E2SF_CML	93230116	80024997	85.84%
	E3SF_CML	90162066	77391694	85.84%
	E7SF_CML	54804260	48074961	87.72%
2DF	E10SF_2DF	53588052	46498827	86.77%
	E5SF_2DF	56203970	49083728	87.33%
	E9SF_2DF	58993438	51256833	86.89%
3DF	E6SF_3DF_100	71087308	63921874	89.92%
	E7SF_3DF_100	63970868	55532503	86.81%
	E8SF_3DF_100	57208682	49587933	86.68%
3DF_50	E3SF_3DF_50	44550608	38454871	86.32%
	E5SF_3DF_50	51239680	44590671	87.02%
	E7SF_3DF_50	56834002	49785038	87.60%
